# Growth hormone-receptor disruption in mice reduces osteoarthritis and chondrocyte hypertrophy

**DOI:** 10.1007/s11357-024-01230-z

**Published:** 2024-06-03

**Authors:** Huanhuan Liu, Trent Davis, Silvana Duran-Ortiz, Tom Martino, Austin Erdely, Shane Profio, Benjamin Osipov, Gabriela G. Loots, Darlene E. Berryman, Patrick M. O’Connor, John J. Kopchick, Shouan Zhu

**Affiliations:** 1 Ohio University Heritage College of Osteopathic Medicine, Ohio University, Athens, Ohio USA; 2 Department of Biomedical Sciences, Ohio University, Athens, Ohio USA; 3 Ohio Musculoskeletal and Neurological Institute (OMNI), Ohio University, Athens, Ohio USA; 4 Edison Biotechnology Institute, Ohio University, Athens, Ohio USA; 5 Diabetes Institute, Ohio University, Athens, Ohio USA; 6 Department of Orthopaedic Surgery, University of California Davis Health, Sacramento, California, USA; 7 Ohio Center for Ecological and Evolutionary Studies, Irvine Hall, Athens, Ohio USA

**Keywords:** Growth hormone receptor, Osteoarthritis, Chondrocyte, Hypertrophy

## Abstract

Excessive growth hormone (GH) has been shown to promote joint degeneration in both preclinical and clinical studies. Little is known about the effect of disrupted GH or GH receptor (GHR) on joint health. The goal of this study is to investigate joint pathology in mice with either germline (*GHR*^*-/-*^) or adult inducible (*iGHR*^*-/-*^) GHR deficiency. Knee joints from male and female *GHR*^*-/-*^ and WT mice at 24 months of age were processed for histological analysis. Also, knee joints from male and female *iGHR*^*-/-*^ and WT mice at 22 months of age were scanned by micro-CT (μCT) for subchondral bone changes and characterized *via* histology for cartilage degeneration. Joint sections were also stained for the chondrocyte hypertrophy marker, COLX, and the cartilage degeneration marker, ADAMTS-5, using immunohistochemistry. Compared to WT mice, *GHR*^*-/-*^ mice had remarkably smooth articular joint surfaces and an even distribution of proteoglycan with no signs of degeneration. Quantitatively, *GHR*^*-/-*^ mice had lower OARSI and Mankin scores compared to WT controls. By contrast, *iGHR*^*-/-*^ mice were only moderately protected from developing aging-associated OA. *iGHR*^*-/-*^ mice had a significantly lower Mankin score compared to WT. However, Mankin scores were not significantly different between *iGHR*^*-/-*^ and WT when males and females were analyzed separately. OARSI scores did not differ significantly between WT and *iGHR*^*-/-*^ in either individual or combined sex analyses. Both *GHR*^*-/-*^ and *iGHR*^*-/-*^ mice had fewer COLX^+^ hypertrophic chondrocytes compared to WT, while no significant difference was observed in ADAMTS-5 staining. Compared to WT, a significantly lower trabecular thickness in the subchondral bone was observed in the *iGHR*^*-/-*^ male mice but not in the female mice. However, there were no significant differences between WT and *iGHR*^*-/-*^ mice in the bone volume to total tissue volume (BV/TV), bone mineral density (BMD), and trabecular number in either sex. This study identified that both germline and adult-induced GHR deficiency protected mice from developing aging-associated OA with more effective protection in *GHR*^*-/-*^ mice.

**Supplementary Information:**

The online version contains supplementary material available at 10.1007/s11357-024-01230-z.

## Introduction

Decline of growth hormone (GH) secretion, or somatopause, is a normal physiologic process of aging. However, dysregulation of GH secretion or its signaling pathway has been shown to cause a myriad of diseases, including diabetes, cancer, osteoporosis, cardiovascular pathology, and arthritis [1, 2]. Some patients with either rheumatoid arthritis (RA) or osteoarthritis (OA) have elevated serum GH but decreased serum IGF-1, when controlling for demographic characteristics such as age and BMI [3], suggesting GH signaling is associated with OA development.

Acromegaly, a disorder related to overproduction of GH, has a high prevalence of arthropathy (~50%) [4]. Characteristic changes to joints in patients with acromegaly include joint space narrowing, osteophytosis, subchondral bone sclerosis, and bone cyst formation. In addition, mutation of the GHR gene in humans due to a deletion of exon 3 (*d3GHR*) is associated with enhancing GH actions [5]. Individuals expressing this mutation exhibit increased prevalence of OA of the hip joint [6, 7]. Conversely, primary GH insensitivity, known as Laron Syndrome (LS), in most cases is due to homozygous inactivating mutation(s) of the GHR gene. The prevalence of OA in LS individuals is not well-known but seems to be decreased relative to their heterozygous siblings (Jaime Guevara, personal communication). Additionally, limited clinical cross-sectional studies indicate that the prevalence of radiographic osteoarthritis (OA) is lower in patients with adult-onset GH deficiency [2, 8].

Genetically modified mouse models with modulated GH actions have offered insight into the role of GH in OA. Mice with over-expression of bovine growth hormone (*bGH*), when compared to littermate controls, have more frequent cartilage clefts, thicker synovium, hypertrophic chondrocytes, subchondral plate thinning, and inflammation [9–11]. By contrast, the role of diminishing GH action in OA remains controversial. For example, the hypopituitary Snell dwarf mice (*dw/dw*) exhibit slower age-associated joint degeneration [12, 13], while *dw/dw* rats exhibit increased severity of cartilage pathology [14]. Our group previously reported that mice with overexpression of GHR antagonist (*GHa*) were protected from aging associated OA [10]. These preclinical studies are in alignment with clinical observations that excessive GH promotes while reduced GH actions limit joint degeneration. However, a recent study using a mouse model of inducible death of GH expressing cells (*AOiGHD*) showed that adult-onset reduction of GH increases OA development [15]. It remains unknown how modulating GH signaling through disruption of GHR affects OA as a function of aging.

The objective of this study is to characterize joint phenotypes of mice with either germline deficiency (*GHR*^*-/-*^) or adult-onset deletion of GH receptor (*iGHR*^*-/-*^). We hypothesize that the absence of GH action due to *GHR* gene disruption decreases OA severity compared to WT mice; genetic studies presented herein strengthen this hypothesis, and offer new insights into modulating GH activity as a potential new therapeutic strategy for OA.

## Methods

### Animal models

*GHR* ^-/-^, *iGHR*^*-/-*^ and WT littermates were propagated in the Edison Biotechnology Institute at Ohio University where they were bred and raised [16, 17]; all animals are on C57BL/6J genetic background. Twenty-four (24) month-old *GHR*^-/-^ (5 males, 5 females) and WT littermates (5 males, 5 females) were used in this study. The *iGHR*^*-/-*^ mice were generated through breeding *B6.129-Gt(ROSA)26Sor*^*tm1(cre/ERT2)Tyj*^*/J* with *Ghr*^*flox/flox*^ mice [18]. For *iGHR*^*-/-*^ mice, 95-100 μl of tamoxifen dissolved in peanut oil was intraperitoneally injected to ablate the *GHR* gene in 12-month-old mice. Age-matched control mice received peanut oil injection. A total dose of 0.32 mg of tamoxifen/g of body weight was administered. Mice received an injection once per day over five consecutive days [17, 19]. The *iGHR*^-/-^ mice and littermate controls at 22 months of age (10 months following GHR deletion) were used for this study. All mouse protocols were approved by Ohio University’s Animal Use and Care Committee.

### Micro-CT (μCT) of mouse knee joints

Whole knees were scanned (SCANCO μCT 35, Bassersdorf, Switzerland) following standard μCT protocols (energy=55 kVp, intensity=114 mA, 15 μm nominal voxel size, integration time=900 ms) for the analysis of rodent bone [20]. Trabecular bone was analyzed at the distal femoral epiphysis. Volumes of interest for the femoral epiphysis included all trabecular bone enclosed by the growth plate, including both medial and lateral condyles. Trabecular regions were designated on each two-dimensional transverse slice using manually drawn contours that excluded the cortical shell. Trabecular bone volume per total volume (Bone Volume Fraction: BV/TV), trabecular thickness (Tb.Th), apparent bone mineral density (Apparent BMD; mg HA/cm3 TV), and other trabecular bone parameters were directly measured using the intrinsic analytical tools.

### Mouse joint histology processing and sectioning

Isolated knee joints were fixed in 4% formaldehyde for 48 hours at 4°C and decalcified in 10% EDTA for 14 days at 4°C. Samples were then dehydrated and embedded intact into paraffin in a standardized anatomical position (e.g., patella facing down with femur and tibia forming equal angles to the rim of the cassette) to facilitate comparable sectioning. Joints were then serially sectioned through the coronal plane at a thickness of 8 μm. Three-to-four thin sections were collected on each glass slide, with five representative mid-coronal sections selected for staining and visualization. Two slides were stained with Safranin-O and subsequently used for OA grading, with three other slides immune-stained for collagen X (COLX) and matrix metalloprotease (ADAMTS-5).

### Safranin O staining

Two slides (approximately 30 μm apart) of the five selected representative midcoronal slides were deparaffinized, rehydrated, and sequentially stained with Fast Green and Safranin-O for bone and cartilage, respectively.

### OA histology grading

Two experienced graders (T.D. and S.P.) evaluated the Safranin-O-stained sections from all joints. Slides were first randomized and assigned a temporary identification code to blind graders to genotype. Sections were then evaluated using both OARSI OA [21] and Mankin grading schemes [22] as shown below (Table 1), and included the following: Articular cartilage structure score, cartilage damage, safranin-O staining score, cartilage matrix, and hypertrophic chondrocytes. All four compartments (lateral femur, medial femur, lateral tibia, medial tibia) were graded independently by each grader. Individual scores from the four compartments were averaged to generate a whole joint score. For the Mankin Grading Scheme, the sum of scores for average Cartilage Damage, average Cartilage Matrix, and average Hypertrophic Chondrocytes were compiled to generate the Mankin Score.

**Table 1:** Modified Mankin grading scheme

Parameter	Grade	Description
Articular Cartilage Structure	0	Normal
	1	Undulating articular surface, but no fibrillation
	2	Mild superficial fibrillation involving < half of plateau/condyle
	3	Mild superficial fibrillation involving ≥ half of plateau/condyle
	4	Mild fibrillation/clefts/loss involving up to 1/3 depth of noncalcified articular cartilage thickness < half of plateau/condyle
	5	Mild fibrillation/clefts/loss involving up to 1/3 depth of noncalcified articular cartilage thickness ≥ half of plateau/condyle
	6	Moderate fibrillation/clefts/loss involving up to 2/3 depth of noncalcified articular cartilage < half of plateau/condyle
	7	Moderate fibrillation/clefts/loss involving up to 2/3 depth of noncalcified articular cartilage ≥ half of plateau/condyle
	8	Severe fibrillation/clefts/loss involving > 2/3 depth of noncalcified articular cartilage thickness < half of plateau/condyle
	9	Severe fibrillation/clefts/loss involving > 2/3 depth of noncalcified articular cartilage thickness ≥ half of plateau/condyle
	10	Clefts/loss of articular cartilage through tidemark
	11	Clefts/loss of articular cartilage through to subchondral bone
Tidemark duplication	0	None (only one tidemark)
	1	2 tidemarks
	2	> 2 tidemarks
	3	No visible tidemark remaining
Safranin-O staining	0	Normal (no loss of staining in non-calcified cartilage)
	1	Moderate loss of staining in up to ½ depth of noncalcified cartilage thickness and involving < half of plateau/condyle
	2	Moderate loss of staining in up to ½ depth of noncalcified cartilage thickness and involving ≥ half of plateau/condyle
	3	Moderate loss of staining in > ½ depth of noncalcified cartilage thickness and involving < half of plateau/condyle
	4	Moderate loss of staining in > ½ depth of noncalcified cartilage thickness and involving ≥ half of plateau/condyle
	5	Severe loss of staining in up to ½ depth of noncalcified cartilage thickness and involving < half of plateau/condyle
	6	Severe loss of staining in up to ½ depth of noncalcified cartilage thickness and involving ≥ half of plateau/condyle
	7	Severe loss of staining in > ½ depth of noncalcified cartilage thickness and involving < half of plateau/condyle
	8	Severe loss of staining in > ½ depth of noncalcified cartilage thickness and involving ≥ half of plateau/condyle
Calcified cartilage	0	0-5 hypertrophic chondrocytes
	1	6-10 hypertrophic chondrocytes
	2	> 10 hypertrophic chondrocytes

### Immunohistochemical (IHC) staining for COLX, ADAMTS-5, and GHR

The remaining three slides (of the five described above) were deparaffinized, rehydrated, and incubated with antigen retrieval R-Buffer A (EMS, 62707-10) at 80 °C for one hour. Slides were then treated with 2% H_2_O_2_, blocked using 5% BSA, and incubated overnight at 4°C with the following primary antibodies: anti-COLX (abcam, #49945, 1:2500 dilution) or anti-ADAMTS-5 **(**ThermoFisher, #PA1-1751A, 1:250 dilution) or anti-GHR (abcam, ab134078). Negative controls were processed in parallel using rabbit IgG. Anti-ADAMTS-5 and anti-GHR staining was detected using goat anti-rabbit antibody conjugated with Horseradish Peroxidase (HRP) (abcam, #6721, 1:1000 dilution); Goat anti-mouse antibody conjugated with HRP (abcam, #6789, 1:1000 dilution) was used for anti-COLX. HIGHDEF Red IHC Chromagen HRP (Enzo, 11141807) was used for color development following manufacturer protocols. Sections were also counter-stained with hematoxylin for 30 seconds. For ADAMTS-5 and GHR, both total chondrocyte number and the number of positively stained chondrocytes in articular cartilage (including both calcified and non-calcified) from all four compartments were counted. The ratio of positively stained chondrocytes was then calculated accordingly for each group (ratio = # of positively stained chondrocytes/total # of chondrocytes). ImageJ Fiji (Johannes Schindelin et al. version 1.2) was used for the semi-quantitative analysis of COLX IHC staining. Images at 20x magnification were uploaded, and any COLX stained area was encircled using the polygon section function. The total cartilage area was then encircled with the polygon section function using concentric lamellae as the delineating marker for subchondral bone. Images were then cropped to only include cartilage for color deconvolution specific to hematoxylin and DAB staining. Both mean and mode DAB staining intensity was measured after minimum and maximum parameters were determined from an average of five samples. Immunohistochemical results were expressed as a ratio of the COLX stained area to the total cartilage area or a ratio of stained chondrocytes to total chondrocytes for ADAMTS-5 and GHR.

### Statistical analysis

An independent sample t-test with unequal variances was used to compare mean scores and ratios for the *iGHR*^*-/-*^ or *GHR*^-/-^ compared to WT littermates for OA and IHC. All scores and ratios were expressed as mean ± 95% SD.

## Results

### GHR^-/-^ mice are protected from developing aging associated OA.

**Fig. 1:**
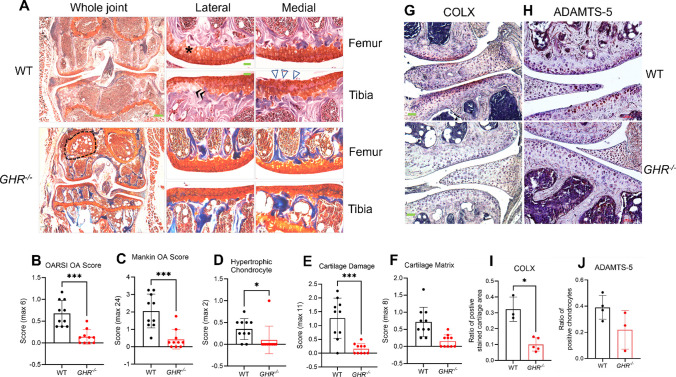
Mice with growth hormone receptor deficiency *(GHR*^-/-^) (24 months) are protected from developing aging-associated OA. (**A**) Safranin-O and Fast Green-stained images of knee joints from WT and *GHR*^*-/-*^ mice. Low-magnification (4x) of the whole joint is on the left, and high-magnification (20x) of each compartment (lateral femur, medial femur, lateral tibia, medial tibia) is on the right. Scale bar (green rectangle) equals 100 μm. Symbols: **Δ**, cartilage loss; *, chondrocyte hypertrophic change, dashed circle lines indicate adipocytes in bone marrow. Quantification of OA severity comparing *GHR*^*-/-*^ (n=10) and WT (n=10) with (**B**) OARSI score, and (**C**) Mankin score. (**D**) Hypertrophic Chondrocyte, (**E**) Cartilage Damage, and (**F**) Cartilage Matrix scores are subcategories in the Mankin score. (**G**) representative immunohistochemical staining for COLX, (**H**) ADAMTS-5*.* (**I**) The average rectangular area of superficial uncalcified cartilage positively stained for COLX*.* (**J**) The ratio of positive-stained ADAMTS-5 chondrocytes to the total count per compartment of the cartilage tissue. *n* = 5 *GHR*^*-/-*^ for COLX and *n* = 3 WT for COLX. Data is shown as mean ± SD

Joint analysis of the 24-month-old mice with germline GHR deficiency (*GHR*^*-/-*^) revealed that the overall joint size was visibly smaller in *GHR*^*-/-*^ mice compared to their littermate WT controls (Fig. 1A, whole joint). Additionally, more lipid droplets were present in the bone marrow of *GHR*^*-/-*^ mice compared to WT controls (Fig. 1A, dashed circle line). Articular cartilage in WT showed typical aging-associated mild to moderate degeneration characterized by superficial fibrillation, loss of proteoglycan staining, and an increase in the number of hypertrophic chondrocytes (Fig. 1A). By contrast, the cartilage surface of *GHR*^*-/-*^ mice was remarkably smooth and evenly stained with proteoglycan Safranin-O. Significantly fewer hypertrophic chondrocytes were visible in *GHR*^*-/-*^ mice (Fig. 1A, higher magnification), as also reflected quantitatively in the significantly lower hypertrophic chondrocyte score in *GHR*^*-/-*^ mice compared to WT (Fig. 1D). Both OARSI (Fig. 1B) and Mankin (Fig. 1C) scores were significantly lower in *GHR*^*-/-*^ compared to WT mice. *GHR*^*-/-*^ mice also had a significantly lower level of cartilage damage compared to WT (Fig. 1E). However, Safranin-O staining intensity (cartilage matrix) only showed a trend to decrease in *GHR*^*-/-*^ mice (Fig. 1F).

**Fig. 2:**
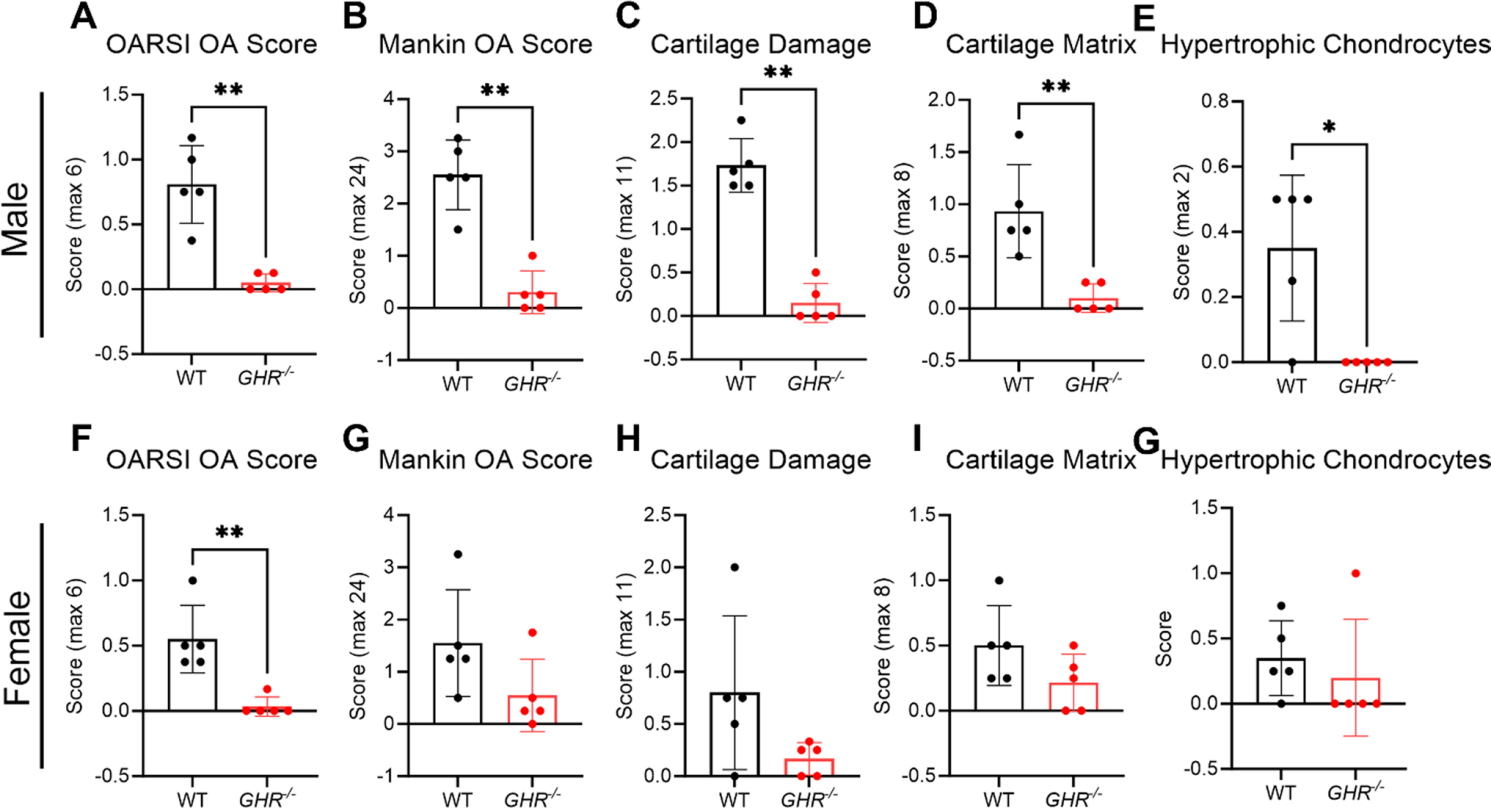
OA pathology scores in WT and *GHR*^*-/-*^ mice separated by male and female groups. (**A-E**) OARSI, Mankin, Hypertrophic Chondrocyte, Cartilage Damage, and Cartilage Matrix scores in WT (*n* = 5) and *GHR*^*-/-*^ (*n* = 5) male mice. (**F-G**) OARSI, Mankin, Hypertrophic Chondrocyte, Cartilage Damage, and Cartilage Matrix scores in WT (*n* = 5) and *GHR*^*-/-*^ (*n* = 5) female mice.

It is well documented that OA presents differently between sexes [23], at both epidemiological and pathophysiological mechanistic levels. In order to constrain potential sex differences in GHR signaling associated OA development, we stratified the OA scores by sex. Although OARSI scores were significant different for both male and female groups (Fig. 2A & F), the Mankin score was only significantly lower in males (Fig. 2B) but not females (Fig. 2G). Similarly, other parameters such cartilage damage (Fig. 2C & H), cartilage matrix (Fig. 2D & I), and hypertrophic chondrocytes (Fig. 2E & G) all showed significantly lower scores in *GHR*^*-/-*^ compared to WT in males but not females. These results indicate potential sex-mediated differences exist regarding the effects of GHR deficiency on OA development in mice.

Additionally, WT and *GHR*^*-/-*^ joints were stained for COLX and ADAMTS-5 protein. The greatest staining density of COLX was in the superficial zone of the uncalcified cartilage with positive staining extending towards the tidemark and the matrix surrounding the hypertrophic chondrocytes (Fig. 2G). WT littermates had significantly greater stained area ratio than *GHR*^*-/-*^ (32.3% vs 10.1%) (Fig. 2J). The staining for ADAMTS-5 was primarily in chondrocyte lacunae and the tidemark with subtle background staining in the deep zone of the uncalcified cartilage (Fig. 2H). There was no significant difference between *GHR*^*-/-*^ and WT mice for the ratio of chondrocytes positively stained for ADAMTS-5 (Fig. 2J).

### iGHR^-/-^ mice are protected from developing aging associated cartilage degeneration in the knee joint.

**Fig. 3:**
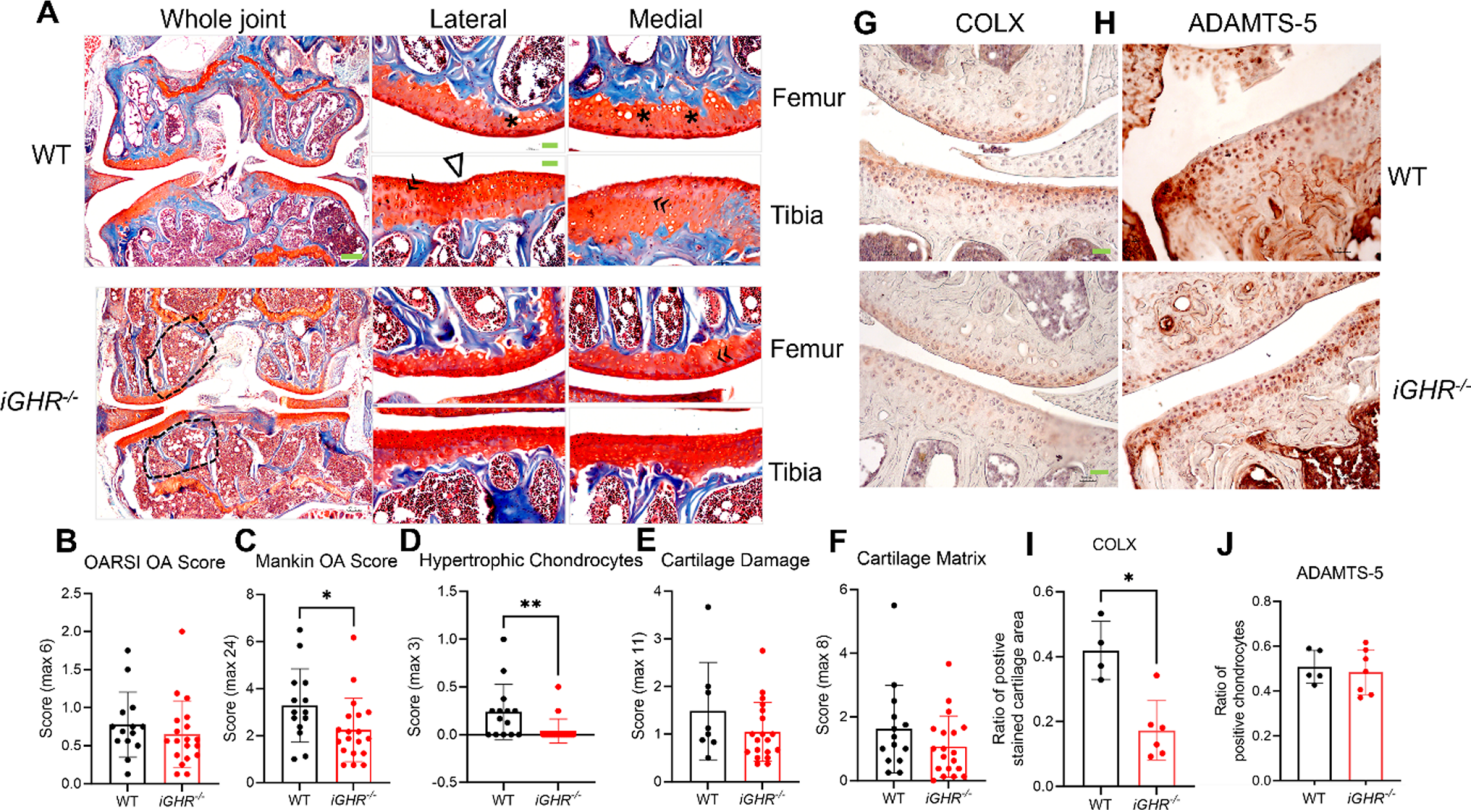
Induced disruption (at 12 months) of growth hormone receptor gene (*iGHR*^*-/-*^) decreases chondrocyte hypertrophy and overall Mankin OA Score at 22 months of age. (**A**) Safranin-O and Fast Green stained-images of knee joints from WT and *iGHR*^*-/-*^ at 22-months of age. Low magnification (4x) of the whole joint is on the left, high magnification (20x) of each compartment (lateral femur, medial femur, lateral tibia, medial tibia) is on the right. Scale bar (green rectangle) equals 100 μm. Symbols: **Δ**, cartilage surface cleft with chondrocyte aggregation; **«**, loss of proteoglycan in cartilage matrix; *, chondrocyte hypertrophic change; dashed circles indicate adipocytes in bone marrow. Quantification of OA severity comparing *iGHR*^*-/-*^ (n=19) and WT (n=14) with (**B**) OARSI score and (**C**) Mankin score. (**D**) Hypertrophic Chondrocyte, (**E**) Cartilage Damage, and (**F**) Cartilage Matrix scores are subcategories in the Mankin score. Representative immunohistochemical staining for (**G**) type X collagen (COLX) and (**H**) matrix metalloprotease (ADAMTS-5) in *iGHR*^*-/-*^ and age-matched WT*. n* = 6 *iGHR*^*-/-*^ for COLX and *n* = 5 WT for COLX. *n* = 7 *iGHR*^*-/-*^ for ADAMTS-5 and *n* = 5 WT for ADAMTS-5. (**I**) The average rectangular area of superficial uncalcified cartilage positively stained for COLX**.** (**J**) The ratio of positive-stained ADAMTS-5 chondrocytes to the total count per compartment of the cartilage tissue*.* Data are shown as mean ± SD. * *p* < 0.05

In order to investigate the effect of GHR deficiency during adulthood on OA, we further analyzed knee joints of age-matched *iGHR*^*-/-*^ (disrupted at 12 months of age) and littermate control mice at 22 months of age. Compared with the WT, there is more than 60% decrease of GHR expression level in the cartilage tissue in *iGHR*^*-/-*^ mice (Supplementary Figure 1). Cartilage on the medial and lateral tibia plateau of control mice had uneven and undulating joint surface, whereas the tibial joint surfaces in *iGHR*^*-/-*^ mice were smooth and flat (Fig. 3A). Chondrocyte aggregation and loss of red proteoglycan staining, both early signs of joint degeneration, were also present at the joint surface of the control mice compared with *iGHR*^*-/-*^ mice (Fig. 3A). Though there was no significant difference in OARSI scores between WT and *iGHR*^*-/-*^ mice (Fig. 3B), the average Mankin score was significantly lower in *iGHR*^*-/-*^ mice (Fig. 3C). We further examined the sub-scores for different categories of the Mankin score. Compared to WT, *iGHR*^*-/-*^ mice had a significantly lower hypertrophic chondrocyte score (Fig. 3D), consistent with the observation that hypertrophic chondrocytes were clustered in the cartilage of WT but not *iGHR*^*-/-*^ mice (Fig. 3A). Other parameters such as cartilage damage (Fig. 3E) and cartilage matrix (Fig. 3F) were not significantly different between control and *iGHR*^*-/-*^ mice. Consistent with previous findings demonstrating a higher whole-body fat content [17], *iGHR*^*-/-*^ mice also exhibit increased lipid in the bone marrow (Fig. 3A, dashed black polygons).

To further evaluate joint degeneration, we stained joints for the chondrocyte hypertrophy marker COLX and the aggrecan-degrading matrix metalloprotease ADAMTS-5. COLX positive staining appeared to be evenly distributed in the superficial non-calcified cartilage zone in WT controls while more concentrated ‘islands’ were found in the chondrocyte lacunae in the calcified zone of *iGHR*^*-/-*^ mice. Notably, more intense staining of COLX was present in the control group compared to *iGHR*^*-/-*^ mice (Fig. 3G), as manifest by a significantly higher COLX stained area ratio of the cartilage (Fig. 3I). In contrast, abundant expression of ADAMTS-5 can be seen in both WT and *iGHR*^*-/-*^ mouse joints (Fig. 3H). Quantification of the ratio of ADAMTS-5 positive stained chondrocytes showed no difference between WT and *iGHR*^*-/-*^ groups (Fig. 3J).

**Fig. 4:**
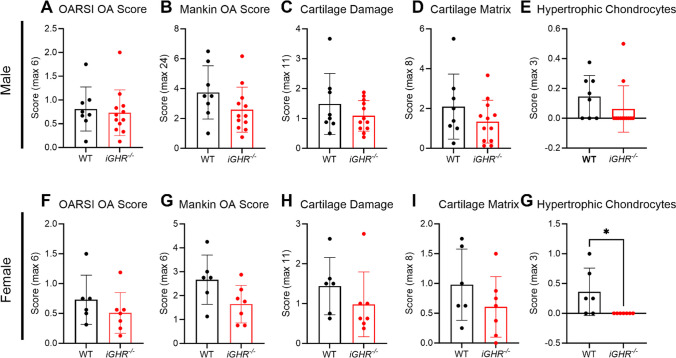
OA pathology scores in WT and *iGHR*^*-/-*^ mice separated by male and female groups. (**A-E**) OARSI, Mankin, Hypertrophic Chondrocyte, Cartilage Damage, and Cartilage Matrix scores in WT (*n* = 8) and *iGHR*^*-/-*^ (*n* = 12) male mice. (**F-G**) OARSI, Mankin, Hypertrophic Chondrocyte, Cartilage Damage, and Cartilage Matrix scores in WT (*n* = 6) and *iGHR*^*-/-*^ (*n* = 7) female mice. * *p* < 0.05

We also stratified the OA scores by sex. No significant sex difference between WT and *iGHR*^*-/-*^ was observed for OARSI (Fig. 4A), Mankin (Fig. 4B), cartilage damage (Fig. 4C), or cartilage matrix (Fig. 4D). Interestingly, female (and not male) *iGHR*^*-/-*^ mice exhibited a significantly lower hypertrophic chondrocyte score compared to WT mice (Fig. 4E & G).

**Fig. 5:**
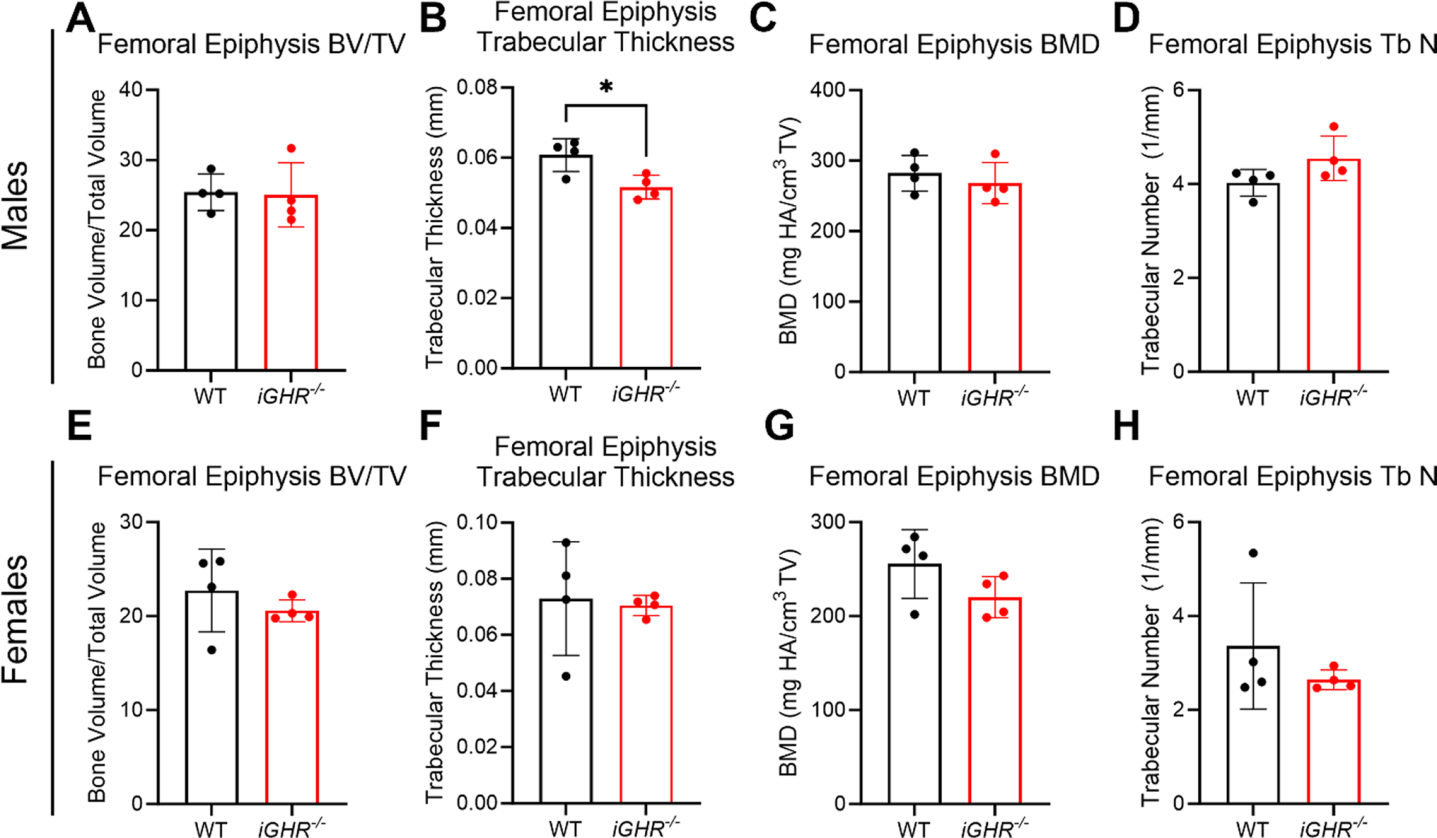
μCT analysis of the subchondral bone in the femoral epiphysis of WT and *iGHR*^*-/-*^ mice reveal a lower trabecular thickness with GHR deficiency. (**A**) Bone volume fraction (BV/TV), (**B**) trabecular thickness, (**C**) bone mineral density (BMD), and (**D**) trabecular number of WT (*n* = 4) and *iGHR*^*-/-*^ (*n* = 4) male mice. (**E**) Bone volume fraction (BV/TV), (**F**) trabecular thickness, (**G**) bone mineral density (BMD), and (**H**) trabecular number of WT (*n* = 4) and *iGHR*^*-/-*^ (*n* = 4) female mice. * *p* < 0.05

Trabeculae in the subchondral bone were visibly thinner in *iGHR*^*-/-*^ mice (Fig. 3A), suggesting a potential effect on bone modeling/remodeling with GHR deficiency. In order to further characterize subchondral bone, we performed a micro-CT (μCT) analysis of the joints. Compared with WT, male (but not female) *iGHR*^*-/-*^ mice had significantly thinner trabeculae in the femoral epiphysis (Fig. 5B & F). Other subchondral bone parameters such as bone volume to total volume (BV/TV; *Bone Volume Fraction*) (Fig. 5A and E), bone mineral density (BMD) (Fig. 5C & G), and trabecular number (Fig. 5D & H) were not different between WT and *iGHR*^*-/-*^ in both sexes.

## Discussion

Growth hormone (GH) is a strong regulator of growth and metabolism and is an FDA-approved drug for the treatment of several clinical disorders including childhood and adult GH deficiency. Additionally, it is commonly used off label as an anti-aging substance [24] due to its anabolic effect. However, illegal or excessive use of GH is well-known to have a myriad of negative health effects including joint pain [25] and the potential to develop diabetes [26] and a variety of cancers [27, 28]. Moreover, it is well documented that patients with acromegaly have accelerated joint degeneration, whereas there is limited knowledge about the prevalence of OA in patients with GH deficiency (GHD) or GH insensitivity (LS). We have recently shown that mice with overexpression of *bGH* develop early onset as well as progressive cartilage lesions, synovitis, and subchondral plate thinning [10]. Conversely, our current study indicates that mice with disrupted GH signaling through either germline or adult induced *GHR* knockout were protected from developing OA during aging, with more effective protection in the germline versus inducible (at 12 months) GHR gene disruption. Our current and previous studies support the conclusion that excessive GH promotes OA development and that decreased GH action (through GHR modulation) protects from OA development.

Numerous animal models with germline disruption of the GH/IGF-1 axis such as in Ames, Snell, *lit/lit*, *GHR*^*-/-*^, and *GH*^*-/-*^ show increased health and lifespan benefits [29]. The *GHR*^*-/-*^ mice, which model individuals with Laron Syndrome, exhibit improved cognition, resistance to diabetes, and reduced neoplasia [30]. These mice also exhibit exceptional longevity and hold the Methuselah mouse prize for the world’s longest-lived laboratory mouse with a lifespan close to 5 years [16], exceeding the typical laboratory mouse lifespan by ~ 2 years. The study conducted herein demonstrates for the first time that *GHR*^*-/-*^ mice are also resistant to developing aging associated knee OA. At 12-24 months of age when WT mice start to develop some level of aging-associated joint degeneration, the knee joint of *GHR*^*-/-*^ mice maintained a smooth joint surface with no sign of cartilage matrix degradation or chondrocyte hypertrophy characteristic of OA onset (Fig. 1). It is possible that *GHR*^*-/-*^ mice have delayed OA development during aging, necessitating an investigation of the knee joint in older *GHR*^*-/-*^ mice. One obvious challenge will be to find appropriate control WT mice at such an advanced age. Another interesting observation from this study relates to sexual dimorphism in the OA phenotype. When stratified by sex, all OA pathology scores were significantly lower in male *GHR*^*-/-*^ compared to WT controls, while only the OARSI score is significantly lower in female *GHR*^*-/-*^ compared to WT in controls. This is generally consistent with numerous reports on sex-specific expression of OA severity in preclinical models [31, 32].

Interestingly, postnatal deletion (at 6 months of age) of *GHR* can retain some but not all the health benefits of congenital *GHR* deficiency, along with sex-specific expression in certain traits. For example, ablation of *GHR* in mice at 6 months of age increased insulin sensitivity only in males whereas lifespan was increased in females but not in males [17]. In our current study, *iGHR*^*-/-*^ mice were less effectively protected from developing OA compared to *GHR*^*-/-*^ mice. We found that *iGHR*^*-/-*^ mice were moderately protected from developing joint degeneration during aging only if males and females were combined in the comparison. Neither OARSI nor Mankin scores were significantly different between WT and *iGHR*^*-/-*^ within the same sex, though female *iGHR*^*-/-*^ mice had a significantly lower score in the hypertrophic chondrocyte subcategory. Importantly, mice with GHR ablation at 12 months of age, as used in this study, have not been comprehensively examined to date, and thus, may exhibit a unique phenotype compared to earlier reports in which GHR disruption occurred at younger ages.

The differences between the *GHR*^*-/-*^ and *iGHR*^*-/-*^ mice are likely due to lack of GHR signaling versus reduction of GH signaling, respectively, at both local and systemic levels considering the GHR gene was deleted constitutively 12 through a ubiquitous *B6.129-Gt(ROSA)26Sor*^*tm1(cre/ERT2)Tyj*^*/J* line in the *iGHR*^*-/-*^ line. In addition, since GH and IGF-1 act in an endocrine and an autocrine/paracrine manner, they share some overlapping physiological functions [33–35]. It is possible that part of effects of GHR deficiency on the joint is through IGF-1 signaling or through other factors such as insulin and adiponectin. We have an on-going effort in lab to delete GHR in the cartilage tissue specifically using the driver *Aggrecan-Cre*^*ERT2*^, which will help us to decipher the relative contribution of local vs systemic effect of decreased GHR signaling.

GH regulates both increased bone size and mass during growth and is considered a major regulator of postnatal body growth [36]. *GHR*^*-/-*^ mice have been reported to have impaired bone growth and reduced BMD in the femur as well as in lumbar vertebrae [37, 38]. Additionally, a recent study reported that GHR deficiency in mice also compromised bone strength through reducing osteocyte lacunar number and increasing lacunar volume [39]. In this study, we are the first to report that GHR deficiency during adulthood (*iGHR*^*-/-*^) reduced trabecular thickness in the femoral epiphysis, a factor that no doubt impacts subchondral bone strength, the local loading environment, and ultimately, the progression joint disease.

GH is also well-known for its role in promoting lipolysis [40]. Indeed, both *GHR*^*-/-*^ and *iGHR*^*-/-*^ mice have a higher percentage of fat mass [17, 41]. This is consistent with our observations that both *iGHR*^*-/-*^ and *GHR*^*-/-*^ have more and larger adipocytes in the bone marrow (Figs. 1A, 3A). We have recently reported that GH can directly alter a wide range of metabolic pathways in chondrocytes, including amino acid metabolism and fatty acid oxidation (FAO) [10]. Since dysregulated metabolism has been increasingly recognized as an important regulator for OA, additional studies to examine metabolic factors specific to cartilage in *iGHR*^*-/-*^ and *GHR*^*-/-*^ mice and their role in mediating OA disease progression is warranted. In this study, reduced load on the joints could potentially contribute to the healthier joint phenotype in both *GHR*^*-/-*^ and *iGHR*^*-/-*^ mice compared to WT. However, emerging evidence shows that the beneficial effect of GHR deficiency could be due to a combination of systemic and local effects. Our ongoing efforts in the lab also include examining how increased or decreased GH actions affect cellular metabolism of joint cells (e.g., chondrocytes) that may also contribute to OA development.

Our group previously identified that mice with over-expression of a GH receptor antagonist (*GHa*) are protected from developing OA [10]. The current work consistently shows that disruption of GH action through deletion of *GHR* also protected mice from developing OA during aging. In fact, the GH receptor antagonist Pegvisomant is an FDA-approved drug used world-wide to treat patients with acromegaly. Taken together, work by our team provides the foundation for future human translational research to test the therapeutic efficacy of inhibiting GH signaling in adult life to treat OA.

Limitations of the study include a small sample size of the *GHR*^*-/-*^ mice after stratification for sex. As a result, the sex-specific effect of *GHR* knockout on OA development was not determined. Other aspects of OA analysis, such as the OA pain behavioral assay and more comprehensive computerized tomography (μCT) of subchondral bone (e.g., proximal tibia, subchondral plate, etc.), are necessary next steps to better characterize joint degeneration during aging. On-going work by our teams investigating the general role and specific mechanisms of GH and GHR in joint health includes experimental designs to overcome these limitations.

In summary, detailed OA pathology grading as well as immunohistochemical analysis of OA associated markers demonstrated that removal of GH action is associated with suppression of chondrocyte hypertrophic changes. Thus, both congenital (*GHR*^*-/-*^) and *iGHR*^*-/-*^ mice are protected from developing aging-associated OA development, with more effective protection in congenital *GHR*^*-/-*^ mice.

### Supplementary information

ESM 1 (DOCX 2321 kb)

## Data Availability

All relevant data are within the manuscript and its Additional files.
