# Supplementary material for: Growth hormone-receptor disruption in mice reduces osteoarthritis and chondrocyte hypertrophy
Source: GeroScience. 2024 Jun 3;46(5):4895–908. doi: 10.1007/s11357-024-01230-z (PMC11336010; doi:10.1007/s11357-024-01230-z)
Supplement: Supplementary file 1 — (DOCX 2321 kb) [file 11357_2024_1230_MOESM1_ESM.docx]

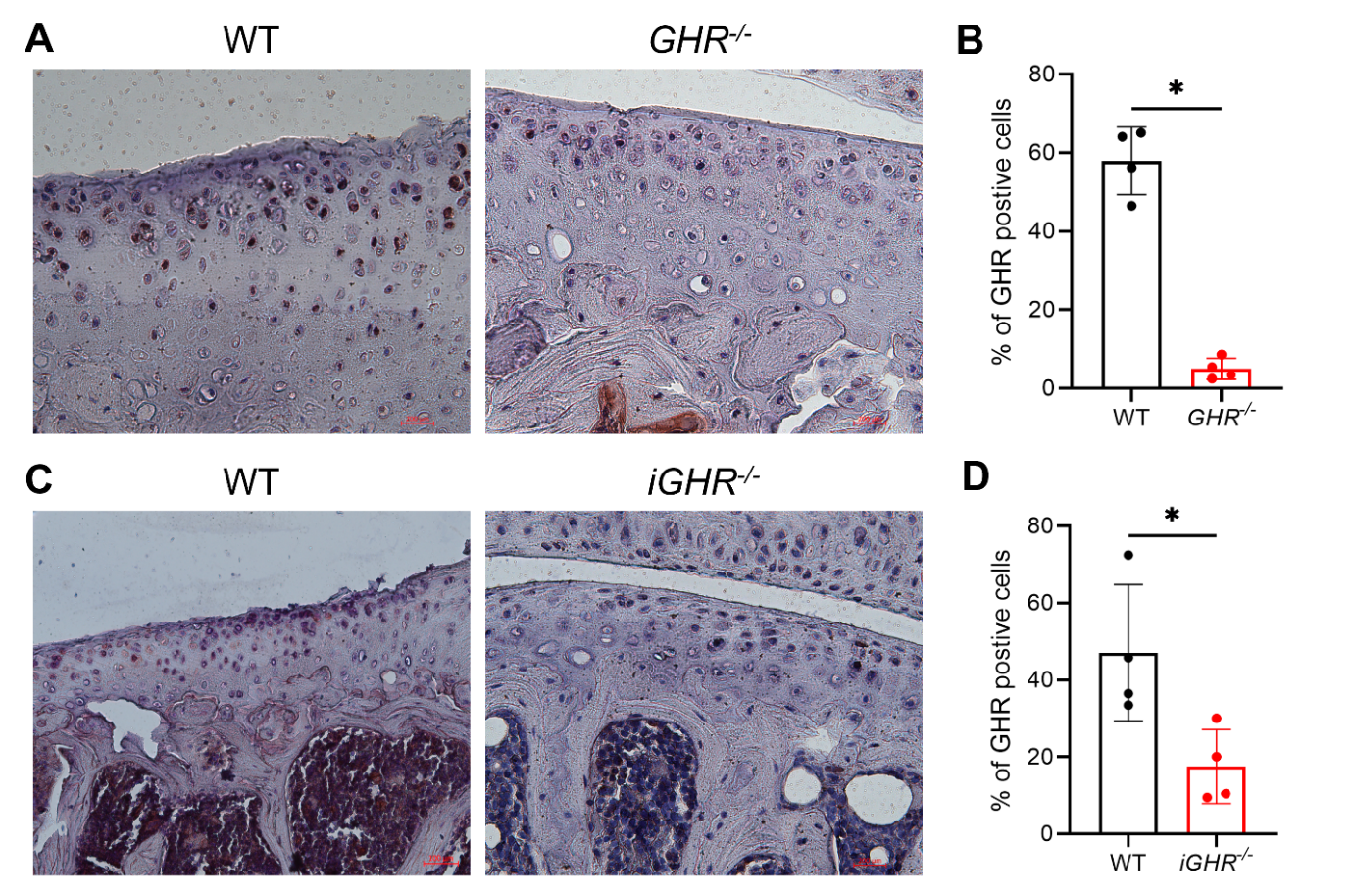


**Supplementary Figure 1. Immunohistochemistry (IHC) staining for GHR in *GHR^-/-^* and *iGHR^-/-^* mice.** (**A & C**) Representative image of IHC staining for GHR in WT and *GHR^-/-^* mice, and WT and *iGHR^-/-^* mice. (**B & D**) Percentage of GHR positively stained chondrocytes. * p < 0.05.
